# Spread and Evolution of Respiratory Syncytial Virus A Genotype ON1, Coastal Kenya, 2010–2015

**DOI:** 10.3201/eid2302.161149

**Published:** 2017-02

**Authors:** James R. Otieno, Everlyn M. Kamau, Charles N. Agoti, Clement Lewa, Grieven Otieno, Ann Bett, Mwanajuma Ngama, Patricia A. Cane, D. James Nokes

**Affiliations:** Kenya Medical Research Institute (KEMRI)–Wellcome Trust Research Programme, Kilifi, Kenya (J.R. Otieno, E.M. Kamau, C.N. Agoti, C. Lewa, G. Otieno, A. Bett, M. Ngama, D.J. Nokes);; Pwani University, Kilifi (C.N. Agoti); Public Health England, Salisbury, UK (P.A. Cane); University of Warwick, Coventry, UK (D.J. Nokes)

**Keywords:** respiratory syncytial virus, viruses, respiratory diseases, RSV, genetic diversity, evolutionary dynamics, G protein, phylogenetic analysis, respiratory infections

## Abstract

In February 2012, the novel respiratory syncytial virus (RSV) group A, genotype ON1, was detected in Kilifi County, coastal Kenya. ON1 is characterized by a 72-nt duplication within the highly variable G gene (encoding the immunogenic attachment surface protein). Cases were diagnosed through surveillance of pneumonia in children at the county hospital. Analysis of epidemiologic, clinical, and sequence data of RSV-A viruses detected over 5 RSV seasons (2010/2011 to 2014/2015) indicated the following: 1) replacement of previously circulating genotype GA2 ON1, 2) an abrupt expansion in the number of ON1 variants detected in the 2014/2015 epidemic, 3) recently accumulation of amino acid substitutions within the ON1 duplicated sequence, and 4) no clear evidence of altered pathogenicity relative to GA2. The study demonstrates the public health importance of molecular surveillance in defining the spread, clinical effects, and evolution of novel respiratory virus variants.

Respiratory syncytial virus (RSV) is a major cause of pneumonia and bronchiolitis among infants and children globally (*1*,*2*). Although immune responses develop in those who have had RSV infection during childhood, these persons remain susceptible to RSV upper respiratory tract reinfection throughout life (*3*). No licensed RSV vaccine exists. Of the 11 proteins encoded by the RSV genome, the attachment glycoprotein (G) is the most variable and has been shown to accumulate amino acid changes over time (*4*). RSV is classified into 2 groups, RSV-A and RSV-B (*5*); each group is divided into genotypes (*6*), and these are further characterized into variants (*7*). Globally, RSV viruses belonging to different groups, genotypes, and variants often co-circulate in epidemics (*7*,*8*). The phenomenon of reinfection and difficulty in developing a vaccine may in part be due to the antigenic diversity and variability in the virus (*9*).

Two novel RSV genotypes with large duplications of amino acids in the attachment G glycoprotein have been detected globally. In 1999, the BA genotype was detected in Buenos Aires, Argentina; the genotype had a 60-nt duplication within the C-terminal region of the G gene (*10*). The BA variant subsequently spread rapidly throughout the world, becoming the predominant group B genotype, and in some regions replacing all previous circulating RSV-B genotypes (*11*). More recently, in December 2010, genotype ON1, with a 72-nt duplication (also within the C-terminal region of the G gene), was detected in Ontario, Canada (*12*). Viruses belonging to this genotype have rapidly spread and diversified globally (*13*–*20*). Such emergent genotypes appear to have a fitness advantage over preceding genotypes of the same RSV group (*21*). Of public health interest is whether increased fitness is associated with increased severity and immune evasion (with potential vaccine modality implications).

The temporal progression of RSV genotypes can be followed directly because of the unique tags (the duplications), which provides a rare opportunity to learn more about the introduction, spread, severity, and related selection processes (including immune evasion) for RSV and to obtain insights into the nature of emergence of novel virus variants. In this regard, we undertook an in-depth analysis of RSV-A genotype ON1 epidemiology in Kilifi, a county in coastal Kenya. In Kilifi, RSV epidemics typically begin during September–November of 1 year and continue until July–August of the following year, with a peak in cases during January–March. We have analyzed sequence data collected over 5 RSV epidemic seasons in Kilifi (2010/2011 to 2014/2015), which includes the period after the initial detection of this novel genotype within Kilifi.

## Materials and Methods

### Study Location and Population

The study was undertaken in Kilifi County and is part of surveillance aimed at understanding the epidemiology and disease effects of RSV-associated pneumonia cases in this region (*22*). Respiratory swab samples (combined nasopharyngeal and oropharyngeal) were collected from September 2010 through August 2015 from children ages 1 day to <5 years admitted to Kilifi County Hospital (KCH) with syndromically defined severe or very severe pneumonia (referred to here as lower respiratory tract infections, LRTIs), as defined in Table 1 and previously (*22*)

**Table 1:** Demographic and clinical characteristics of cases of pneumonia caused by respiratory syncytial virus A genotypes ON1 and GA2 in children admitted to Kilifi County Hospital, September 2010–August 2015*

Characteristic	Genotype, no. (%)		
	ON1	GA2	Total
Age, y			
<1	223 (85.4)	158 (87.3)	381 (86.2)
>1	38 (14.6)	23 (12.7)	61 (13.8)
Sex			
F	118 (45.2)	70 (38.6)	188 (42.5)
M	143 (54.8)	111 (61.3)	254 (57.5)
Cough			
No	5 (1.9)	4 (2.2)	9 (2.0)
Yes	256 (98.1)	177 (97.8)	433 (98.0)
Breathing difficulty			
No	15 (5.8)	3 (1.7)	18 (4.1)
Yes	246 (94.3)	178 (98.3)	423 (95.9)
Chest wall indrawing			
No	6 (2.3)	6 (3.3)	12 (2.7)
Yes	255 (97.7)	175 (96.7)	430 (97.3)
Inability to feed			
No	211 (81.2)	165 (91.2)	376 (85.3)
Yes	49 (18.9)	16 (8.8)	65 (14.7)
Oxygen saturation, %			
>90	214 (82.0)	144 (79.6)	358 (81.0)
<90	47 (18.0)	37 (20.4)	84 (19.0)
Prostration/unconsciousness			
No	240 (92.0)	172 (95)	412 (93.2)
Yes	21 (8.0)	9 (5.0)	30 (6.8)
Pneumonia status			
Severe†	203 (77.8)	137 (75.7)	340 (76.9)
Very severe‡	58 (22.2)	44 (24.3)	102 (23.1)
Hospital stay, d			
1–4	160 (62.0)	101 (55.8)	261 (59.4)
>4	98 (38.0)	80 (44.2)	178 (40.5)
Outcome			
Survived	250 (96.9)	177 (97.8)	427 (97.3)
Died	8 (3.1)	4 (2.2)	12 (2.7)

*The denominator was 442 samples but for some of the features it ranged from 439 to 442 †[Cough or difficulty in breathing] *and* chest wall indrawing ‡[Cough or difficulty in breathing] *and* hypoxic or prostrate/unconscious.

### Study Samples and Laboratory Procedures

All specimens were screened for RSV by 2 methods (*22*–*24*). Raw samples were tested for RSV antigen by immunofluorescence antibody test (Chemicon International Inc., Temecula, CA, USA). Viral RNA was extracted from respiratory samples using QIAamp Viral RNA Mini Kit (QIAGEN, Hilden, Germany) and tested for RSV (differentiating groups A and B) by multiplex real-time reverse transcription PCR. All RSV-positive samples by either test were taken forward for processing. In addition, a small number of RSV-negative samples were sequenced.

The viral RNA was reverse transcribed into cDNA by using the Omniscript RT Kit (QIAGEN). The cDNA was then amplified with primers targeting the G ectodomain region (*3*,*25*), and the amplicons were sequenced by using BigDye version 3.1 chemistry on an ABI 3130xl (Applied Biosystems, Waltham, MA, USA). Sequence reads were assembled into contigs by using Sequencher version 5.0.1 (Gene Codes Corp., Ann Arbor, MI, USA). The sequences analyzed here have been deposited in GenBank (accession nos. KX453303–KX453534); previously reported sequences from Kilifi added to this analysis had also been deposited in GenBank (accession nos. KF587911–KF588014) (*13*).

### Global Comparison Dataset

To determine the relatedness of the Kilifi viruses to those circulating around the world and thereby clarify their global context, we downloaded all RSV-A G-gene sequences collected during 2010–2015; deposited in GenBank as of January 19, 2016; and 241 nt to 687 nt in length. A total of 995 sequences from 24 countries were used in this analysis. For the whole dataset and for some countries, we further grouped sequences by calendar year for temporal analysis. We subsampled unique sequences (sequences that differed by >1 nt from any other sequence over the sequenced region) by epidemic season (Kilifi only) or per calendar year.

### Sequence Alignments and Diversity Analysis

All sequences, from Kilifi and the global dataset, were collated and aligned using MAFFT (multiple alignment using fast Fourier transform) alignment software version 7.272 (*26*). We calculated the variability of nucleotides and amino acids using MEGA 6.06 (*27*).

### Phylogenetic Analyses

We used MEGA 6.06 to infer maximum-likelihood phylogenetic trees under the general time reversible model with the site heterogeneity gamma model (*27*). This model was the best substitution model as determined by IQ-TREE version 1.4.2 (*28*). Bootstrapping with 1,000 iterations was implemented to evaluate branch support of the phylogenetic clusters. We assigned RSV-A genotypes as previously determined by Peret et al. (*6*) and Eshaghi et al. (*12*). To position the genotype ON1 viruses in the global context, we examined ON1 lineages as recently assigned by Duvvuri et al. (*20*).

### RSV-A Variants Analysis

We determined the number of genotype GA2 and ON1 variants circulating in Kilifi and globally using a recently developed pragmatic criterion (*7*,*8*). In brief, a variant is a virus or a group of viruses within a genotype that possesses ≥4 nt differences in the G ectodomain region when compared with other viruses. This analysis was done using usearch v8.1.1861 (*29*).

### Protein Substitution and Selection Analysis

The *N*-glycosylation sites were predicted by using the NetNGlyc 1.0 server (*30*). We only considered the default Asn-X-Ser/Thr sequon (when X was not proline) for prediction. We also analyzed for patterns of change in amino acids using python scripts. Finally, we looked for potential positively selected and co-evolving sites using the Datamonkey server (http://www.datamonkey.org/). For positive selection analysis, we used 3 methods: SLAC (single likelihood ancestor counting), FEL (fixed effects likelihood), and MEME (mixed effects model for evolution).

### Statistical Analyses

We explored associations between demographic, clinical, or outcome variables and RSV genotypes for all cases of RSV-positive severe and very severe pneumonia. We used logistic regression computing odds ratios (ORs) in Stata version 13 (StataCorp LP, College Station, TX, USA).

## Results

Over the 5 RSV epidemics examined (2010/2011 to 2014/2015), a total of 4,010 samples were collected from eligible children; 3,561 (88.8%) were tested for RSV and 881 (24.7%) RSV-positive samples were identified (Technical Appendix Table 1). Of these samples, 600 (68.1%) were RSV-A. The G gene was successfully sequenced in 442 (73.7%) samples. An additional 41 sequences were available from samples that were negative by both immunofluorescent antibody test and PCR or from patients with mild pneumonia (data for these cases were not included in the clinical severity analysis). Thus, we carried 483 sequences for phylogenetic analysis. The sequences ranged from 618 nt to 690 nt in length, corresponding to nucleotides 295–912 of the reference strain A2 (M74568).

**Figure 1:**
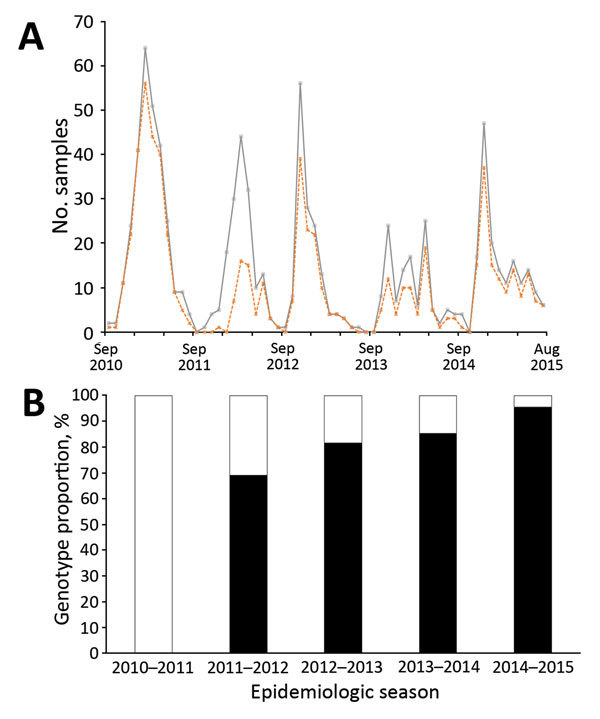
Circulating patterns of respiratory syncytial virus (RSV) in Kilifi, Kenya, September 2010– August 2015. A) Total RSV-positive cases (gray continuous line) and typed RSV-A samples (dotted orange line) by month. B) The proportion of RSV-A genotypes ON1 (black) and GA2 (green) per epidemic season. An RSV epidemic season is designated to start in September of 1 year until August of the following year. Unusually, for the last 3 seasons, group A represents most of all RSV cases

We found that 2 RSV-A genotypes were circulating in Kilifi: ON1 (n = 283, 58.6%) and GA2 (n = 200, 41.4%). The temporal prevalence of the total RSV, RSV-A, and genotypes ON1 and GA2 is shown in Figure 1 and Technical Appendix Table 1. We observed rapid replacement of the previously circulating dominant GA2 genotype by ON1 in Kilifi, from a prevalence of 0% in the 2010/2011 epidemic to a prevalence of 67.4% in 2011/2012 when ON1 was first detected in Kilifi, and to 96.1% in the recent 2014/2015 epidemic. In addition, RSV-A predominated in 3 consecutive RSV epidemics from 2012/2013 to 2014/2015.

To investigate the demographic and clinical effects of RSV-A genotype ON1 in Kilifi, we compared the proportions of GA2- and ON1-infected case-patients by sex, age, clinical features of cough, difficulty in breathing, chest wall indrawing, inability to drink, hypoxia, prostration/consciousness, pneumonia status (severe or very severe pneumonia), length of hospital stay, and death at the hospital (Table 1). The proportions of both genotypes were very similar for the demographic and clinical characteristics analyzed. However, the proportion of patients with ON1 infection who were unable to eat was more than double that for GA2-infected case-patients (18.9% vs. 8.8%), and this difference was found significant by logistic regression (OR 2.40, 95% CI 1.31–4.36; Table 2). Nonetheless, the proportion with very severe pneumonia was no higher in ON1 infections than in GA2 infections (OR 0.89, 95% CI 0.57–1.39).

**Table 2:** Clinical severity comparison between cases of pneumonia caused by respiratory syncytial virus A genotypes ON1 and GA2 in children admitted to Kilifi County Hospital, September 2010–August 2015*

Characteristic	Unadjusted odds ratio (95% CI)	p value
Age <1 y	0.85 (0.49–1.49)	0.579
Male sex	0.76 (0.52–1.12)	0.172
Clinical features		
Cough	1.16 (0.31–4.37)	0.830
Breathing difficulty	0.28 (0.08–0.97)	0.045
Chest wall indrawing	1.46 (0.46–4.59)	0.520
Inability to feed	2.40 (1.31–4.36)	0.004
Oxygen saturation <90%	0.86 (0.53–1.38)	0.521
Prostration/unconsciousness	1.67 (0.75–3.74)	0.211
Pneumonia status, very severe	0.89 (0.57–1.39)	0.609
Hospital stay, >4 d	0.77 (0.53–1.14)	0.192
Outcome, died	1.416 (0.42–4.78)	0.575

*The denominator was 442 samples but for some of the features it ranged from 439 to 442.

**Figure 2:**
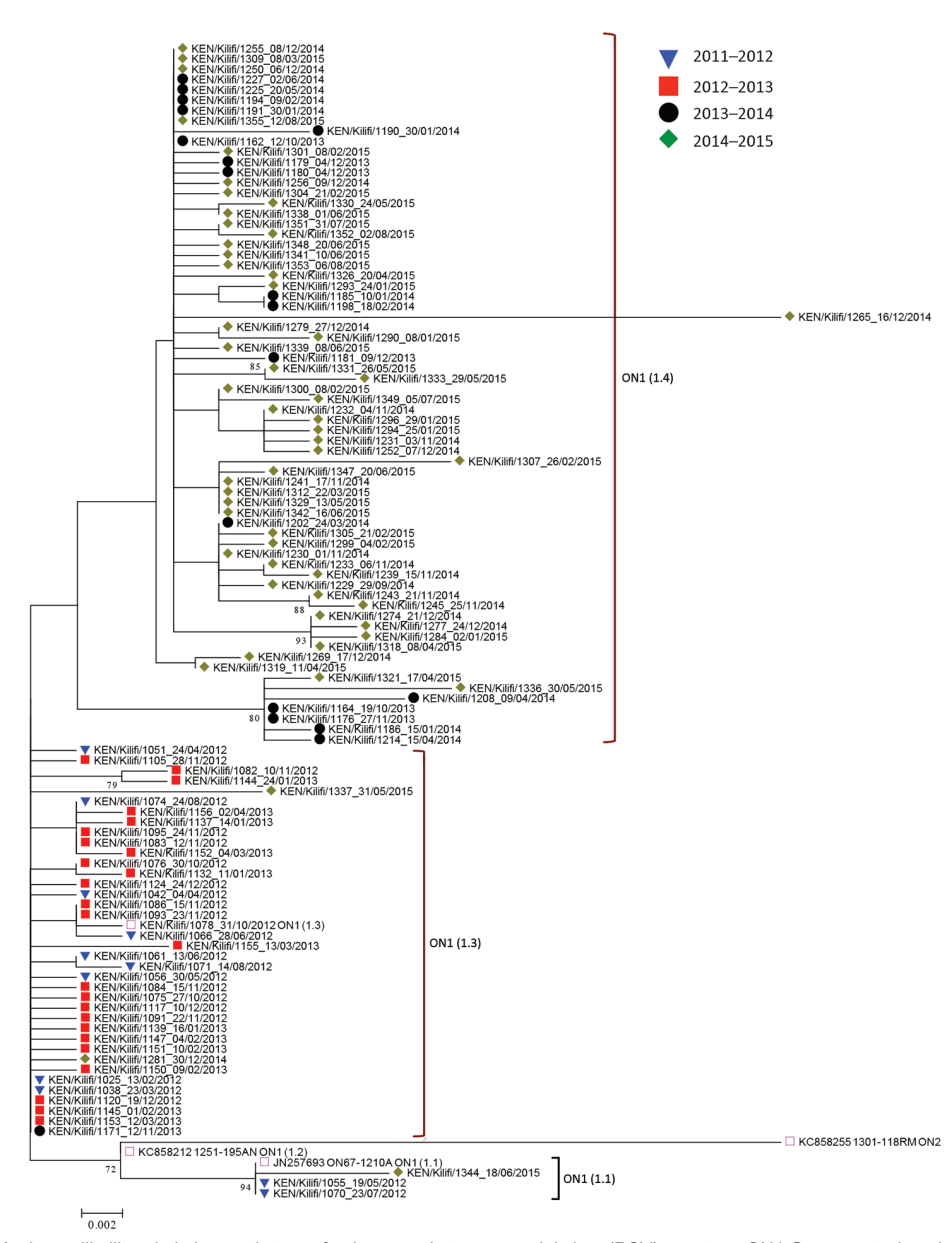
Maximum-likelihood phylogenetic tree of unique respiratory syncytial virus (RSV) genotype ON1 G gene ectodomain sequences from Kilifi, Kenya, 2012–2015. The taxa are color coded by the epidemic season of detection (key), and the names represent KEN/Kilifi/serialnodate of collection. Note that although the study detected RSV ON1 in the epidemic season 2011/2012, the first ON1 cases were in 2012. Scale bar indicate nucleotide substitutions per site.

The maximum-likelhood tree shows the clustering of unique genotype ON1 sequences in Kilifi (Figure 2). Two genotype ON1 lineages recently defined by Duvvuri et al. (*20*) are shown to be circulating in Kilifi: lineages ON1 [1.1] and ON1 [1.3]. Of these 2 lineages, ON1 [1.3] was the most prevalent in 2011/2012 and 2012/2013. However, a potential new lineage, denoted here as ON1 [1.4], clustered away from ON1 [1.3] and seemed to have recently arisen comprising sequences from the strains circulating in the 2013/2014 and 2014/2015 epidemics. The genetic divergence (p distance) between ON1 [1.4] and the other ON1 lineages identified in Kilifi ranged from 0.013 to 0.045, similar to the genetic distances between the previously defined ON1 lineages.

We detected a total of 66 RSV-A variants during the entire surveillance period in Kilifi (Table 3). The variants comprised 1–82 sequences; 39 (59.1%) of the 66 variants were singletons. Most variants did not persist between epidemics (46/66 [69.7%]). However, 14 variants persisted for 2 consecutive seasons, 1 for 4 consecutive seasons, and 5 for 2 nonconsecutive seasons. Therefore, the number of variants (accumulated by epidemic) increased to 86 by 2014/2015. The number of GA2 variants declined consistently, from 17 variants in 2010/2011 (before ON1 arrived) to only 4 variants in 2014/2015. On the other hand, the number of ON1 variants assigned remained at 5 variants between 2011/2012 and 2012/2013 before increasing to 8 variants in 2013/2014, then rising markedly to 25 variants in 2014/2015.

**Table 3:** Temporal occurrence of respiratory syncytial virus A (RSV-A) variants (rows) detected in Kilifi, Kenya, 2010/2011–2014/2015

Variant*	Genotype	Epidemic (no. variants/epidemic)								
		2010/2011		2011/2012		2012/2013		2013/2014		2014/2015
		17		15		13		12		29
1	ON1									
2	ON1									
3	ON1									
4	ON1									
5	ON1									
6	ON1									
7	ON1									
8	ON1									
9	ON1									
10	ON1									
11	ON1									
12	ON1									
13	ON1									
14	ON1									
15	ON1									
16	ON1									
17	ON1									
18	ON1									
19	ON1									
20	ON1									
21	ON1									
22	ON1									
23	ON1									
24	ON1									
25	ON1									
26	ON1									
27	ON1									
28	ON1									
29	ON1									
30	ON1									
31	ON1									
32	ON1									
33	ON1									
34	ON1									
35	GA2									
36	GA2									
37	GA2									
38	GA2									
39	GA2									
40	GA2									
41	GA2									
42	GA2									
43	GA2									
44	GA2									
45	GA2									
46	GA2									
47	GA2									
48	GA2									
49	GA2									
50	GA2									
51	GA2									
52	GA2									
53	GA2									
54	GA2									
55	GA2									
56	GA2									
57	GA2									
58	GA2									
59	GA2									
60	GA2									
61	GA2									
62	GA2									
63	GA2									
64	GA2									
65	GA2									
66	GA2									

*A variant was defined as a group of viruses (when group includes a singleton) within a genotype that possesses ≥4 nt differences in the G ectodomain region than other viruses (see Methods). Dark blue, GA2; green, ON1.

Seven codon sites were predicted to be *N*-glycosylated within the G protein for the Kilifi sequences: 4 sites for genotype ON1 viruses (codons 103, 135, 237, 318) and 6 sites for genotype GA2 viruses (codons 103, 135, 237, 251, 273, 294). However, none of the potential *N*-glycosylation sites occurred within the 72-nt duplication of the ON1 viruses. Notably for GA2 viruses, sites 237 and 273 were mutually exclusive: a virus belonging to this genotype had either these sites potentially *N*-glycosylated but not both (Technical Appendix Figure 1). 

**Figure 3:**
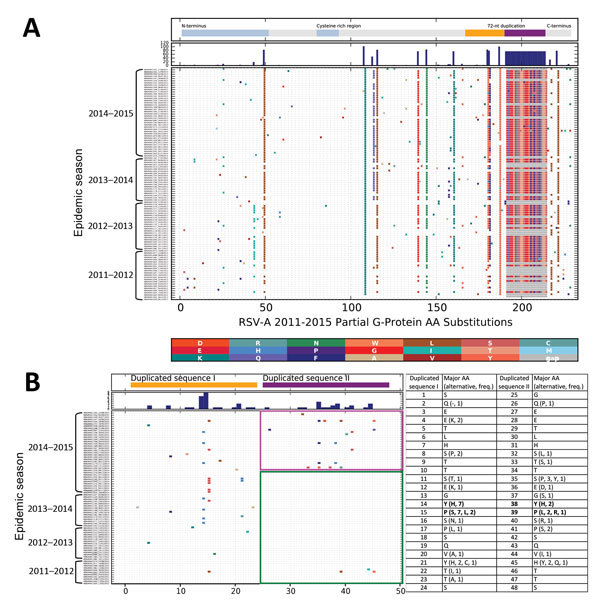
Amino acid substitutions in respiratory syncytial virus A (RSV-A) G protein for sequences isolated in Kilifi Kenya from season 2011/2012 to 2014/2015. All unique protein sequences per epidemic were collated, aligned and the amino acid differences from the earliest sequence determined and marked with vertical colored bars, with the substituted amino acid residue color coded as shown by the key between panels A and B. A) Full aligned aa sequence inferred from the G gene sequences (ON1 and GA2); B) (ON1 only) focuses on the region of the ON1 duplication. The positions shown at the bottom of panels A and B are relative to the first amino acid of the regions analyzed, i.e., from amino acid positions 94 and 260, respectively, of the reference strain A2 (Ref: M74568). Indicated at the top of these panels are the functional domains of the G protein (panel A) and the 72-nt duplication of genotype ON1 (panel B; duplicated sequence I in “orange” and duplicated sequence II in “purple”). Below this, the histogram indicates the total number of changes at each position. C) Concurrent aa positions within the duplicated sequences I and II, and the respective aa substitutions (numbering similar to positions in panel B).

The nucleotide variability and amino acid variability over the 4 seasons are shown in Technical Appendix Table 2. Amino acid substitutions over the sequenced portion of the G protein are shown in Figure 3, panel A. Two codon positions possessed amino acid substitutions that distinguished between ON1 (232G, 253K) and GA2 (232E, 253T) viruses. In addition, the new ON1 [1.4] lineage viruses seem to have fixed a threonine (I/T136T) and acquired a unique substitution (P206Q) that distinguishes them from the other ON1 lineages.

Figure 3, panels B and C, illustrates amino acid substitutions within the duplication region of the Kilifi ON1 viruses. We designated the first set of 72 nt as duplication sequence I and the second set as duplication sequence II. Within this region, we observed that over the 3 seasonal epidemics from 2011/2012 to 2013/2014 and early (September–November) in the 2014/2015 epidemic, amino acid substitutions only occurred within the duplicated sequence I except for 3 substitutions in 2 viruses within the duplicated sequence II. Beginning in December 2014, however, we found numerous substitutions in duplicated sequence II with 2 adjacent and corresponding positions between the duplicated sequences I and II acquiring similar amino acid substitutions (i.e., Y273H and Y297H, P274L/S and P298L/R). Furthermore, sites 273 and 297 were detected to be co-evolving from the Spidermonkey analysis (http://www.datamonkey.org/). However, only 1 ON1 codon site (251) was identified to be positively selected with p<0.05 by >1 method (SLAC, FEL, or MEME).

Using global datasets for genotypes ON1 and BA, we compared the temporal detection of RSV variants within each of these genotypes during the first 5 and 10 years, respectively, from initial detection (Technical Appendix Table 3). We observed an explosion of new ON1 variants globally, from 8 variants in 2011, to 78 variants in 2012, to 153 variants in 2013. However, the number of ON1 variants decreased in 2014 and 2015, which corresponded with a substantial decrease in both the number of ON1 sequences available in GenBank and the countries that have deposited sequences from these years. On the other hand, the number of BA variants seemed to follow a stepwise or punctuated pattern, whereby the number of variants was stable at 1–6 during 1996–2001, dramatically increased and stabilized at 20–30 variants during 2002–2004, and again sharply rose to 82 variants in 2005 (Technical Appendix Table 3). At the country level (Technical Appendix Table 4), the rapid rise in the number of ON1 variants detected in Kilifi was also observed in the Philippines and Germany, although the number of BA variants detected in some of the countries sampled remained relatively stable over time.

## Discussion

We provide a detailed analysis on the spread and the associated demographic, clinical, and evolutionary characteristics of the novel RSV-A genotype ON1 in Kilifi. ON1 was first detected in Kilifi in February 2012, and within that RSV epidemic (2011/2012), it displaced the previous dominant genotype GA2 by attaining a prevalence of 67%. Its dominance has continued to rise to 96% within a span of 4 epidemics. This rapid rate of replacement is unlike previous replacement rates in the same location; for example, when GA5 was displaced by GA2, it took GA2 ≈7 years to reach a prevalence of 95% (*8*). ON1 seems to possess a fitness advantage over GA2. If such fitness is the result of immune evasion, this characteristic has potential implications for vaccines to deliver population level immunity by herd protection (*31*).

We found evidence that infections caused by RSV ON1 are more severe than those caused by GA2, showing a higher prevalence of patients’ inability to eat. However, overall numbers of cases of very severe pneumonia were equal for both genotypes. The data, therefore, do not provide a strong indication of more severe disease arising from the ON1 variant. Duvvuri et al. (*20*) reported significant association of RSV ON1 infection with female patients, which was not evident in our study. Yoshihara et al. (*32*) reported that cases of upper respiratory infection caused by RSV ON1 in Vietnam caused were significantly associated with clinically severe manifestations of wheezing, tachypnea, and difficulty in breathing compared to infections caused by RSV NA1, whereas Panayiotou et al. (*33*), on the contrary, reported that children infected with RSV ON1 in Cyprus experienced significantly milder illness than those infected with RSV GA2. Some studies have reported no differences at all between infections with these genotypes. The discordant results may arise from methodologic differences in analyses, clinical disease definitions, and study designs, chance effects resultant from inadequate sample sizes, differences between viruses in different locations, or even host/environmental differences. Prospective studies specifically designed to evaluate virulence or clinical differences between genotypes may offer more reliable insight.

We noted a change in the alternation of RSV subgroup dominance pattern in Kilifi since ON1 was introduced into this community. While ON2 was replacing GA2, it also appeared to also exclude group B strains. RSV-A predominated over RSV-B in 3 consecutive epidemics from 2012/2013 to 2014/2015. Previously, according to data collected during 2002–2012 in Kilifi, RSV-A predominated in up to 2 consecutive epidemics (*8*). However, it is unclear whether this is a direct effect of ON1, a general change in RSV epidemiologic patterns, or a chance occurrence.

Globally, the prevalence of ON1 seems to vary by location. In Ontario, Canada, where ON1 was first detected in December 2010, the prevalence of ON1 has remained stable at 11%–13% (*20*). Other countries that have similarly reported ON1 prevalence rates <20% include South Africa (*34*) and China (*35*). Reports from Italy (*14*), South Korea (*36*), United States (*17*), Malaysia (*37*), Japan (*19*), Thailand (*16*), Latvia (*38*), and Cyprus (*33*) indicate varied RSV ON1 prevalence of 20%–70%. A recent article reported that ON1 was the sole (100%) RSV-A genotype in Buenos Aires, Argentina, in 2014 (*39*). The varying prevalence suggests that even though ON1 is rapidly spreading globally, host or ecologic differences may determine RSV spread. However, the conclusions of host and ecological differences potentially driving varying prevalence rates in different countries may have been confounded by inadequate/short surveillance periods in these countries.

As shown here and in previous research from Kilifi (*7*,*8*), RSV epidemics are composed of multiple variants, each differing sufficiently to suggest separate introductions into the community (as opposed to arising from diversification during the epidemic). In addition, these variants often do not persist between epidemics, which suggests that each year 1) variants generate local herd immunity, leading to their demise, thus requiring reintroductions; or 2) that many invading variants compete in seeding new seasonal epidemics, and that the preceding year variants lose out (perhaps on a chance basis or as stated above because they are less fit due to variant specific immunity).

RSV accumulates amino acid changes over time (*4*), and we have similarly observed accumulation of amino acid changes in the Kilifi ON1 viruses. It is of interest that in the first 3 RSV epidemic seasons in which we detected ON1 in Kilifi, amino acid substitutions were almost always restricted to the duplicated sequence I of the 72-nt duplication. However, in the 2014/2015 epidemic a virtual explosion in amino acid substitutions was observed within the duplicated sequence II that coincided with a surge in the number of detected ON1 variants. In addition, similar amino acid substitutions occurred in 2 adjacent and corresponding sites in the duplicated sequences I and II, with 1 set of these sites co-evolving. The longer attachment protein of the 72-nt duplication in ON1 viruses appears to offer more opportunities for variable changes and thus greater diversity and increased fitness over previous group A genotypes.

Two codon sites, 232 and 253, within the G protein region analyzed were found to distinguish between genotype ON1 and GA2 viruses. The amino acid change Glu-232-Val has been reported for RSV-A escape mutants that result in loss of reactivity to a specific monoclonal antibody (*4*). Furthermore, a functional analysis of the 60-nt duplication in BA strains has shown that the duplicated region in the G protein of these viruses augment their fitness (*21*). While a similar analysis has not been reported for ON1, it is plausible that the increased fitness observed in ON1 is largely due to the 72-nt duplication. However, G-protein *N*-glycosylation seems to play no role in the increased fitness of ON1 as similar potential *N*-glycosylation codon sites were detected in both ON1 and GA2 and no additional *N*-glycosylation sites were detected within the ON1 duplication region.

The rapid diversification of ON1 observed in Kilifi seems to reflect rapid expansion at the global level. While sampling variability may play a role, and similar to varying prevalence, there was variability in the diversification of ON1 viruses in different countries. The number of ON1 variants seemed stable in some countries (e.g., Japan) while expanding in others (e.g., Germany and Philippines). The temporal distribution of BA variants in different countries, however, was mostly stable. Comparisons in the temporal patterns of genotypes BA and ON1 variants may highlight differences between the RSV group B and A viruses. Increased sampling and surveillance will help illuminate on whether such inter-genotypic and RSV group differences are due to ecologic differences or variable sampling.

In conclusion, it is evident that genotype ON1 is not only rapidly spreading globally but also fast evolving. The result is the near exclusion of the previous dominant group A GA2 genotype. The implications of this apparent increased fitness of RSV-ON1 have yet to be resolved. There is some evidence for increased severity of the virus but this is by no means clear or consistent across studies. Continued surveillance for cases together with collection of detailed standardized clinical data are warranted. The possibility exists that ON1 and other similar new RSV variants (e.g., the BA genotype) gain dominance by evading host immunity. It is reasonable to assume this could lead to evasion of future vaccine induced protection, lessening the herd immunity potential of vaccination, similar to influenza A vaccines.

Technical Appendix Details from analysis of samples collected from eligible children during RSV epidemics in Kilifi, Kenya and amino acid alignment and a maximum likelihood tree highlights the 2 codon sites (237 and 273) on the G protein.
