# Supplementary material for: Spread and Evolution of Respiratory Syncytial Virus A Genotype ON1, Coastal Kenya, 2010–2015
Source: Emerg Infect Dis. 2017 Feb;23(2):264–71. doi: 10.3201/eid2302.161149 (PMC5324789; doi:10.3201/eid2302.161149)
Supplement: Technical Appendix — Details from analysis of samples collected from eligible children during RSV epidemics in Kilifi, Kenya and amino acid alignment and a maximum likelihood tree highlights the 2 codon sites (237 and 273) on the G protein. [file 16-1149-Techapp-s1.pdf]

Article DOI: http://dx.doi.org/10.3201/eid2302.161149

# Spread and Evolution of Respiratory Syncytial Virus A Genotype ON1 Coastal Kenya, 2010–2015

## Technical Appendix

**Technical Appendix Table 1.** Frequency of LRTI inpatient cases, samples tested, total RSV and RSV-A cases, and number sequenced over five successive epidemics in Kilifi, Kenya*

| Epidemic season* | No. LRTI eligible cases | No. (%) samples tested† | No. (%‡) RSV-positive samples | No. (%§) RSV-A samples | No. (%) RSV-A sequences# | No. (%¶) ON1 sequences | No. (%¶) GA2 sequences |
|---|---|---|---|---|---|---|---|
| 2014/2015 | 876 | 866 (98.9) | 203 (23.4) | 133 (65.5) | 128 | 123 (96.1) | 5 (3.9) |
| 2013/2014 | 722 | 576 (79.8) | 124 (21.5) | 74 (59.7) | 68 | 58 (85.3) | 10 (14.7) |
| 2012/2013 | 659 | 563 (85.4) | 142 (25.2) | 107 (75.4) | 87 | 71 (81.6) | 16 (18.4) |
| 2011/2012 | 814 | 718 (88.2) | 151 (21.0) | 54 (35.8) | 46 | 31 (67.4) | 15 (32.6) |
| 2010/2011 | 939 | 838 (89.2) | 261 (31.1) | 232 (88.9) | 154 | 0 (0) | 154 (100) |
| Total | 4,010 | 3,561 (88.8) | 881 (24.7) | 600 (68.1) | 483 | 283 (58.6) | 200 (41.4) |

*Epidemic designated 1st September of one year to 31st August of the following year. RSV, respiratory syncytial virus; LRTI, lower respiratory tract infection.
†As a proportion of the eligible LRTI inpatient cases.
‡As a proportion of the samples tested.
§As a proportion of the RSV positive samples.
#Includes 41 sequences from IFAT/PCR negativeve or mild pneumonia cases.
¶As a proportion of the RSV-A sequences.

**Technical Appendix Table 2.** Nucleotide and amino acid variability in RSV-A genotypes identified in Kilifi, Kenya, 2010/2011 to 2014/2015

| | All RSV-A | | ON1 | | GA2 | |
|---|---|---|---|---|---|---|
| Epidemic season* | Nucleotide | Amino acid | Nucleotide | Amino acid | Nucleotide | Amino acid |
| 2014/2015 | 0.88 | 1.40 | 0.68 | 1.10 | 2.02 | 4.12 |
| 2013/2014 | 1.24 | 2.07 | 0.55 | 0.75 | 1.72 | 3.29 |
| 2012/2013 | 1.21 | 2.14 | 0.46 | 0.58 | 1.69 | 3.13 |
| 2011/2012 | 1.45 | 2.99 | 0.43 | 1.02 | 1.56 | 2.91 |
| 2010/2011 | 0.66 | 0.99 | – | – | 0.66 | 0.99 |
| Total* | 2.04 | 3.41 | 0.85 | 1.23 | 1.01 | 1.71 |

*Epidemic designated from September 1 of 1 year to August 31 of the following year. RSV, respiratory syncytial virus.

**Technical Appendix Table 3.** Frequency of global genotypes BA and ON1 variants detected, by calendar year, from sequences deposited in Genbank*

| BA | | | | ON1 | | | |
|---|---|---|---|---|---|---|---|
| Year | Variants | Sequences | Countries | Year | Variants | Sequences | Countries |
| 1998 | 2 | 4 | 2 | 2010 | 1 | 5 | 2 |
| 1999 | 3 | 11 | 4 | 2011 | 8 | 57 | 7 |
| 2000 | 2 | 2 | 1 | 2012 | 78 | 368 | 19 |
| 2001 | 6 | 23 | 4 | 2013 | 153 | 432 | 15 |
| 2002 | 20 | 51 | 9 | 2014 | 59 | 256 | 8 |
| 2003 | 30 | 60 | 10 | 2015 | 10 | 81 | 2 |
| 2004 | 30 | 144 | 12 | | | | |
| 2005 | 82 | 290 | 13 | | | | |
| 2006 | 101 | 205 | 16 | | | | |

Data extracted from GenBank on January 19, 2016, length 241 to 687 bases.

**Technical Appendix Table 4.** Frequency of genotypes BA and ON1 variants, by calendar year, detected from countries around the world. Sequences deposited in Genbank by the respective countries were used*

| | | Year and variants | | | | | | | |
|---|---|---|---|---|---|---|---|---|---|
| Genotype | Country | 2010 | 2011 | 2012 | 2013 | 2014 | 2015 | | |
| ON1 | Philippines | – | – | 7 | 29 | – | – | | |
| | Germany | – | – | 5 | 26 | – | – | | |
| | Japan | – | – | 5 | 9 | 7 | 1 | | |
| | Spain | – | – | 5 | 7 | 14 | - | | |
| BA | | 1999 | 2000 | 2001 | 2002 | 2003 | 2004 | 2005 | 2006 |
| | Belgium | 2 | – | 5 | 5 | 5 | 5 | 8 | 9 |
| | Argentina | 1 | – | – | 6 | 8 | 5 | – | |
| | Japan | – | – | 1 | 2 | 4 | 3 | 9 | 6 |

*Data extracted from GenBank on  January 19, 2016, length 241 to 687 bases. –, not available.

Publisher: CDC; Journal: Emerging Infectious Diseases
Article Type: Research; Volume: 23; Issue: 2; Year: 2017; Article ID: 16-1149
DOI: 10.3201/eid2302.161149; TOC Head: Research

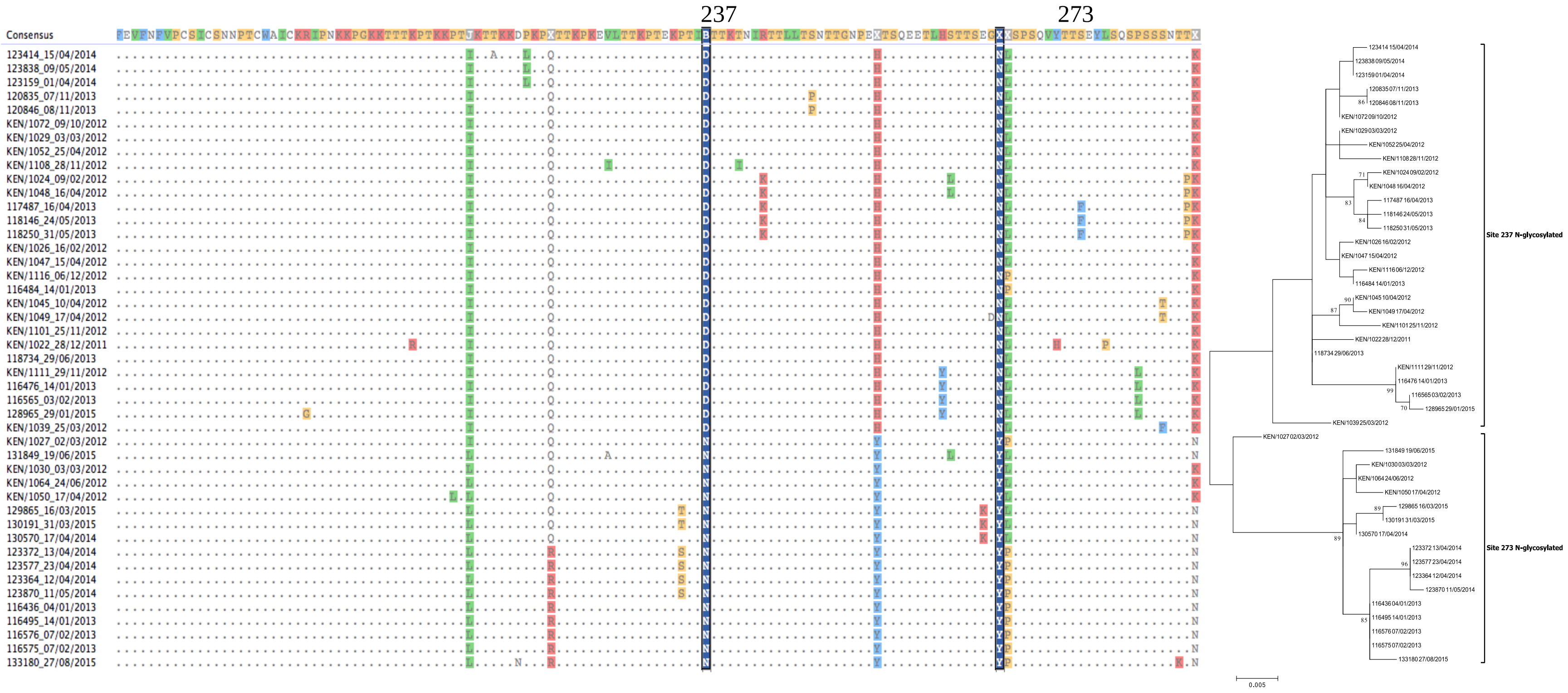


**Technical Appendix Figure 1.** An amino acid alignment and a maximum likelihood ML tree highlighting the 2 codon sites (237 and 273) on the G protein that define 2 groups of GA2 viruses based on *N*-glycosylation patterns. Samples are from Kilifi County Hospital patients, ages 1 day to >5 years, 2012–2015. Scale bar indicates nucleotide substitutions per site.

**Technical Appendix Figure 2.** Amino acid substitutions in respiratory syncytial virus A (RSV-A) G protein for sequences isolated in Kilifi Kenya from season 2011/2012 to 2014/2015. All unique protein sequences per epidemic were collated, aligned and the amino acid differences from the earliest sequence determined and marked with vertical colored bars, with the substituted amino acid residue color coded as shown by the key between panels A and B. A) Full aligned aa sequence inferred from the G gene sequences (ON1 and GA2); B) (ON1 only) focuses on the region

of the ON1 duplication. The positions shown at the bottom of panels A and B are relative to the first amino acid of the regions analyzed, i.e., from amino acid positions 94 and 260, respectively, of the reference strain A2 (Ref: M74568). Indicated at the top of these panels are the functional domains of the G protein (panel A) and the 72-nt duplication of genotype ON1 (panel B; duplicated sequence I in “orange” and duplicated sequence II in “purple”). Below this, the histogram indicates the total number of changes at each position. C) Concurrent aa positions within the duplicated sequences I and II, and the respective aa substitutions (numbering similar to positions in panel B).
